# Acknowledgment to the Reviewers of *Toxins* in 2022

**DOI:** 10.3390/toxins15020096

**Published:** 2023-01-19

**Authors:** Toxins Editorial Office

**Affiliations:** MDPI AG, St. Alban-Anlage 66, 4052 Basel, Switzerland

High-quality academic publishing is built on rigorous peer review. *Toxins* was able to uphold its high standards for published papers due to the outstanding efforts of our reviewers. Thanks to the efforts of our reviewers in 2022, the median time to first decision was 15 days and the median time to publication was 36 days. Regardless of whether the articles they examined were ultimately published, the editors would like to express their appreciation and thank the following reviewers for the time and dedication that they have shown *Toxins*:
Aaron K. HoshideKrishna KantAbdel Razzaq Al-TawahaKristóf Endre KárolyAbdelazeem M. AlgammalKui ZhangAbdeslam AsehraouaKun-Che ChangAbdolrahman KhezriKwang-Soo ShinAbdul QayyumKyle M. BenowitzAbhinandan ChowdhuryKyu-Ho YiAbraão Almeida SantosLaetitia KoppeAbraham Méndez-AlboresLaifeng LuAdam OkorskiLajos ZsomAdam SchrumLarelle FabbroAdela Lopez De CerainLasse LindahlAdnan KhanLato PezoAdnan MustafaLaura Domínguez DíazAdolfo PerrottaLaura Dorina DinuAdrian Florin SpacLaura EscriváAdrian ManLaura KellyAdrian RothenfluhLaura RighettiAdriana Cristina UrcanLaura Soler-VascoAdrianne N. EdwardsLaura ValdezAdrijana LeonardiLaura VicenteAgata PiecuchLaurent DufosséAgnė Laučytė-CibulskienėLaurent MetzingerAgnieszka BossowskaLauro Velazquez-SalinasAgnieszka Klimkowicz-PawlasLavinia RutaAgnieszka PszczółkowskaLeandro Licursi De OliveiraAgnieszka SynowiecLeda GuzmánAgnieszka WnukLee KatsAgostino CasapulloLee Seong WeiAgustín AriñoLei LiuAgustin Melo-CarrilloLei LuoAhmad AlshannaqLei ZhaoAhmed Al-SabiLeijiane F. SousaAhmed BadrLélia ChambelAhmed ElbestawyLenka ŠindlerováAhmed El-KhatibLeo Kei IwaiAhmed M. Abd-ElGawadLeonel PereiraAhrar Ahmad KhanLeticia Diez-QuijadaAibo WuLetizia PolitoAihua LiLevent ÖzçakarAitor RementeriaLiang ChenAkbar HossainLi-Chun HoAkihiko YamamotoLicia PantanoAkio NakashimaLidia BłaszczykAkos MesterhazyLígia Isoni AuadAlberto MantovaniLihua ChenAlden EstepLiliana J. G. SilvaAldo LaganàLingjing JinAldo NicosiaLinn HoffmannAleix CasesLira GaysinaAlejandra Alonso-CalveteLisa VaillancourtAleksandra BocianLjilja TorovićAleksandra SzydłowskaLoïc RambaudAleksei Vorob'evLong-Sen ChangAlessandra DubbiniLonnie Van ZylAlessandra DurazzoLorena Durán-RiverollAlessandra Frances CimbaloLothar BreckerAlessandra RicelliLourival PossaniAlessandro de SireLuana IzzoAlessandro FeolaLuca RuiuAlessia FabbriLucia De CastelliAlessio Filippo PeritoreLucia GambacortaAlessio PaladiniLucía García-OrtegaAlex ArtyukhinLucia Helena FaccioliAlexander G. DvoretskyLucía SoliñoAlexander HawlitschkaLuciana Daniela LarioAlexandra Cristina MunteanuLuciana TartaglioneAlexandra Duarte SilvaLuciano PinottiAlexei SolovchenkoLucie HasoňováAlfonso NarváezLucilene Delazari Dos SantosAlfredo BerardelliLucio MarinelliAli BoolaniLucyna KozlowskaAlicia BoltLudovic MartinelleAlina PlenisLuigi Alberto PiniAlix PierronLuigi CastaldoAllan Rod MerrillLuigi RussoAlma Vázquez-DuránLuís AbrunhosaAmalia StefaniuLuis Carlos MartínezAmandine CaruanaLuís Gustavo Romani FernandesAmar PatelLuis Roberto De Camargo GonçalvesAmer DababatŁukasz StępieńAmir Mohammad MalvandiŁukasz TomczykAmir Sasan Mozaffari NejadŁukasz ŻielonkaAmira AbugomaaLynda PartridgeAmira MoawadLyudmila DimitrovaAmit SrivastavaMª Carmen Louzao OjedaAmit TripathiMa Cristina Del Rincón-CastroAmnart PoapolathepMaciej ChęcińskiAmro AmaraMaciej GajęckiAna AguadoMaciej JarzebskiAna Beatriz Furlanetto PachecoMaciej WoźnyAna BorotaMadalina-Gabriela IliescuAna Da SilvaMagali ReyAna Gago-MartínezMagdalena Dziagwa-BeckerAna Isabel Álvarez-MercadoMagdalena GajęckaAna Isabel OliveiraMagdalena Markowicz-PiaseckaAna Isabel PrietoMagdalena Mazur-KuśnirekAna Leonor Abrahão NencioniMagdalena Polak-ŚliwińskaAna María BotanaMagdalena SobieskaAna Maria UdreaMagdalena ToporowskaAna MornarMagdalena WójciakAna Paula Frederico Rodrigues Loureiro BracarenseMagdalena ZaborowskaAna V. Espinel-IngroffMahendran SekarAnabela M. AlmeidaMahima VediAnahi Elena Ada BucchiniMahmoud F. SeleimanAnant DeshwalMaike KrauseAnas ShamsiMaiko UmemuraAndrás SzabóMaja Šegvić KlarićAndré Lopes FulyMakoto HasegawaAndré Mariz De AlmeidaMakoto TsunodaAndrea BolognesiMałgorzata JarończykAndrea CerratoMalgorzata OlejnikAndréa Emilia Marques StinghenMalihe Mehdizadeh AllafAndrea MarcinnòManas SahooAndrea Roxanne J. AnasMandar JogAndrea SantamatoManuel B. AguilarAndreas BallotManuel Córdoba-DiazAndreas H. LaustenManuel Jiménez-TenorioAndreas VilcinskasManuela ZadravecAndreea Iren SerbanMarc KoyackAndrei NiculaeMarc L. D. de BuyzereAndreia FreitasMarc MarescaAndrew A. WinklerMarcel AmichotAndrew PadulaMarcel BermudezAndrew PoveyMarcelino GutierrezAndrew TurnerMarcin ArciszewskiAndrey V. SybachinMarcin OżarowskiAndrzej KoniecznyMarcin RóżewiczAndrzej SlominskiMárcio F. SimãoAndy PickettMarco Carnevale MiinoAngel CataldiMarco GerdolÁngel L. GuerreroMarco IammarinoAngel YanagiharaMarco InvernizziAnggit SunarwidhiMarcus ScottiAnita GiglioMarek TchórzewskiAnna AndreouMarek WiergowskiAnna BaldisserottoMargarita FernandezAnna Baturo-CiesniewskaMargarita StankovaAnna CampanatiMari Yotsu-YamashitaAnna CzarneckaMaria A. AnderssonAnna Klara MocovaMaria Adele GiamberardinoAnna Kowalik-KlimczakMaría Ángela OlivaAnna KozakMaría Ángeles LezcanoAnna KozirógMaria Angeles RojoAnna M. Kurek-GóreckaMaria Antonietta RagusaAnna MadejskaMaria BelousovaAnna MilczarekMaria Chiara GattoAnna NieróbcaMaría Del Mar García-SuárezAnna PetruczynikMaria Del Rosario IglesiasAnna ShepelyakovskayaMaria Drussilla BuscemiAnna SieroslawskaMaria Elena MarcocciAnna V. GoncharenkoMaria FazzariAnnabel GuichardMaria GeorgiadouAnne Blanc-PotardMaria Giulia BattelliAnne NekarisMaría J. AndradeAnnika KruseMaria Joao SilvaAnthony SaviolaMaria Laura RamirezAnthony WenndtMaria Leonor FaleiroAnthony ZammitMaria Leonor NunesAnton A. NizhnikovMaria MaistoAntonella VirgilioMaría Moreno-del ÁlamoAntonello SantiniMaria Rosaria De MiglioAntonina SorokanMaria-Giuliana VannucchiAntonio BevilacquaMarian BurduceaAntonio Gonzalez-CasadoMariana CastroAntónio NogueiraMariana S. CastroAntonio PortugalMarie-Helene MarionAnwar HussainMarie-Laure Pinel-MarieApostolos N. ApostolidisMarija KovačApurva LadMarijana SokolovicArakere C. UdayashankarMarijn SpeeckaertAram MegighianMariko MiyazakiArancha Llama-PalaciosMarilene DemasiArif SiddiquiMarina AboalArin MarchesiMarina P. IsaevaAristidis TsatsakisMario MasielloArmando A. Rodríguez AlfonsoMario RendaArmando VenancioMarion EhrichArmel GalletMarios KrokidisÁrpád AmbrusMarisa MegnaArto PulliainenMaristella MastoreArturo Edgar Zenteno-GalindoMarius Gustav BredellArunas RamanaviciusMariusz SikoraAshutosh BahugunaMariusz ŚlachcińskiAsif ShajahanMark D. BakerAthanasios AlegakisMark S. McClainAudrey Roy-LachapelleMark W. SumarahAurelio ScavoMark WeaverAurora MontaliMark WilkinsonAxel TouchardMarkus KalkumAyman MahmoudMarkus NaumannAziz KarakayaMarlena SzalataBal SinghMarlene Andrade-GuelBamisope Steve BamisileMarlous FockerBaohua ChenMarta HerreraBarbara BlackwellMarta JarczewskaBarbara CanonicoMarta Waliszewska-ProsółBarbara Illowsky KarpMartha M. EiblBarbara NovakMartin Christian MichelBarbara Pawlik-SkowrońkaMartin PtokBarbara WiewióraMartin SvobodaBarry RosenMartina GagliotiBart De GeestMartina LoiBartlomiej TroczkaMartina TorricelliBaruch SnehMasahiro HiramaBasem S. ZakariaMasamitsu HyodoBaskaran Stephen InbarajMasamitsu MaekawaBeata MessyaszMasanori AritaBeatriz RegueraMasashi MizunoBeatriz Sevilla-MoránMasayo KushiroBei ZhangMasayuki SatakeBen Cristofori-ArmstrongMassimiliano ChettaBen De Lacy CostelloMassimo CorsaliniBen GreenMassimo LucariniBenedito C. PrezotoMateescu CarmenBenito Gomez-SilvaMatias PasqualiBenito Soto-BlancoMatt LewinBenjamin MaskreyMatthew E. ColdironBeob Gyun KimMatthew LebarBernard PoulainMatthew LewinBernát GáborMatthias D. HoferBernd LeplowMatthias EckhardtBernd RodemannMatthias KochBiagio SantellaMatthias PairederBing-Sin LiuMattia RapaBiresaw AberaMaurits K. A. van SelmsBlagoy UzunovMaurizio BrigottiBogdan LewczukMaurizio MulasBogdan SlencuMaurizio NordioBojan ŠarkanMauro Cesar Palmeira VilarBojan ŽunarMaxim BurkinBo-Young HongMehtap P. KaraBradley A. SpicerMeiji Soe AungBrady R. CunninghamMelinda SzilágyiBreda Jakovac-StrajnMelisa Bénard-ValleBrendan M. DugganMeng LuoBrigitte G. DornerMengfei HoBrînduşa Alina PetreMercedes Vázquez-EspinosaBruna CarbasMervi KanervaBruno TiloccaMia EeckhoutBryan Grieg FryMicaela ÁlvarezByung-Kwan SeoMichael AppellC. Benjamin NamanMichael C. PetrielloCalin Mircea GhermanMichael CalcuttCamelia TulcanMichael E. VaydaCansu İlke KuruMichael G. WellerCarlo ColosimoMichael GotsbacherCarlos Alberto CañasMichael J. GermainCarlos Alfredo Gómez BravoMichael L. PretterklieberCarlos Augusto MalmannMichael PoteserCarlos CamposMichael TellierCarlos GarcíaMichail DiakosavvasCarlos MenesesMichał Da̧browskiCarlos RosadoMichal NiemczakCarmen BarcenaMichał NowickiCarmen RubioMichał PiegzaCarmen SolcanMichał ZłochCarmen ValeMichel J. RouxCaterina MorciaMichela MitchellCaterina PeggionMichele VecchioCatherine E. VrentasMichelle S. MostromCecilia TottiMieszko WieckiewiczCelia Maria SoaresMiguel Ángel Sogorb SánchezCelia Marisa Rizzatti-BarbosaMiguel Ponce-VargasCésar MatteiMiguel Rebollo-HernanzCesare AccinelliMihai CenariuCesare MontecuccoMiho JeongCesareo Saiz JimenezMiklós MézesChang ChenMiklós PoórChanglong ShuMilva PepiChangseon RyuMin SunChao-Yu GuoMing Hsien TsaiCharles AffourtitMingyu YeCharles Odilichukwu R. OkpalaMingyuan WangChengmiao YinMiodrag LazarevićChenlin HuMiran BrvarChetan KeswaniMireille FouillaudChetan PaliwalMirko CucinaChian Ju JongMirko FilippettiChiao-Yin SunMiroslav JuráčekChiara BrulloMiroslava KačániováChiara La TorreMirta MilićChien-Che HungMitchell BrinChih-Chuan LinMohamed F. AbdallahChi-Kong ChanMohamed G. ShehataChing-Hu ChungMohamed HassanChinnasamy RagavendranMohamed Jawed AhsanChitra MandyamMohamed Z. M. SalemChi-Tun TangMohammed Rizman-IdidCho Yeow KohMonica FariaChoo Hock TanMonica NegreaChris GarnhamMonika BeszterdaChris MaragosMonika Ehling-SchulzChristian MüllerMontamas SuntravatChristian U. OeingMorana JaganjacChristian WongMrutyunjay JenaChristoph ReinhardtMulunda MwanzaChristopher CoteMunawar IqbalChristopher William HollandMurray B. IsmanChumporn SoowannayanMurugesan ChandrasekaranChung-Cheng WangNadezhda SachivkinaCiro ImbimboNadia YerkovichClaudia FoersterNadja HellmannClaudia RibeiroNakane AkioClaudinéia Pereira CostaNanqin GanClaudio CaprariNaomasa OshiroClaus P. W. ZebitzNark-Kyoung RhoClaus VoglNataliya GruntenkoClemence RougeauxNatraj KrishnanConstanza CardenasNatsuo UedaCorinne AmarNaveen VankadariCristina Gabriela GrigoraşNeal M. DaviesCristina Juan GarcíaNeil AudsleyCristina Nicoleta CiureaNelson SimõesCristina Pinedo-RivillaNicholas Faure WalkerD. Cordoba-DiazNicholas J. PerainoDabor ResiereNick BrandehoffDale R GardnerNii Korley KorteiDalia UrbonavičienėNikola HodkovicováDalibor ŠatínskýNikola MarakovićDamien HaberlinNina KudumijaDana Maria CopoloviciNina RecekDanh C. VuNishiyama-Jr MiltonDaniel GilletNobumichi KobayashiDaniel KamińskiNoor Baity SaidiDaniel TietzeNoureddine BouaïchaDaniel TurudićNuankanya SathirapongsasutiDaniel ValleraNúria FontanalsDaniela CalinaOadi MatnyDaniela IannazzoOctavian Tudorel OlaruDaniela Manila BianchiOhad MazorDaniela NovickOlga A. GoryachevaDaniela Petrova Georgieva-KlisarovaOlga A. KoksharovaDaniele MercatelliOlga A. SinitsynaDapeng PengOlga BelykhDarryl R. WoodOlga Meiri ChaimDauri J. TessmannOlga OrlovaDave WunschelOlga P. GavrilovaDavid DoraOlga V. EfremenkovaDavid Hernández HerreroOlimpia LaiDavid Lozano-PaniaguaOluwatobi KolawoleDavid NelsenOrly Laskar-LevyDavid PereiraOrnella RossettoDavid PerrinOsamu ArakawaDavid RileyOsmindo R. Pires JúniorDavid SergeevichevOvidiu OnigaDavide LonatiPablo EstevezDavinia PlaPablo MirallesDawid GawlińskiPanagiota KatikouDebasish Kumar DeyPanagiota XaplanteriDebra EhrlichPanagiotis A. EliopoulosDelavar ShahbazzadehPanagiotis TassisDennis BideshiPang-Chui ShawDennis E. JewellPaola RoncadaDetlef H. KrieterPaolo NataleDiana AntalPaolo TortoraDiana HendrickxParamanandham KrishnamoorthyDiana ObistioiuParisa GazeraniDiego CotellaPasquale RussoDiego MartinezPatricia CasinoDiego San MauroPatricia Hernández-MartínezDiego SaukaPatricia Losada-PerezDietrich K. HofmannPatricia RegalDimitrios SklirosPatricio DíazDing WangPatricio Orellana-PalmaDirk DresslerPatrick DansetteDmitry KhochenkovPatrick G. ArndtDomenico Azarnia TehranPatrick Michael McNuttDomenico DavolosPatrick SchlievertDomenico FrancoPatrizia LicataDomenico TortorellaPaucean AdrianaDominique KouaPaula C. B. PaígaDong-Zong HungPaula RodriguesDonna HillPaulo Marcos PintoDorin IonescuPaulo ValeDorota Gołąbek-SulejczakPavan KumarDragan MilicevicPavel BabicaDrago BesloPaweł GlibowskiDuc-Cuong BuiPawel KafarskiĐuro JosićPawel KwiatkowskiEdgar CambazaPawel PohlEdmund MaserPaweł ŚwitEdwin J. Vazquez-CintronPedro Abreu-MendesEfi LevizouPedro Infante-CossioEhsan Moghaddas KiaPedro Martínez-GómezEiichi KumamotoPedro Reis-CostaEiichi SatoPeiqiang MuEiji ArakawaPeiqiang YuEijiro AkasakaPeng LiaoEikan MishimaPeramaiyan RajendranEkaterina ChernovaPeta HarveyEkaterina N. LyukmanovaPeter S. SpencerEkaterina V. GrizanovaPeter V. DubovskiiEkaterini Simões GoudourisPetr HenebergEkram AliasPetr KarlovskyEl Hassan AjandouzPhilipp EllerEleftherios TouloupakisPhilipp HessElena BonciuPhilippe HuberElena EfremenkoPhilippe-Henri SecretanElena LeychenkoPia Lopez-JornetElena Rodica IonescuPierluigi RevegliaEleonora NuceraPierre RougeEleonóra SpekkerPierre TufféryEleonore FröhlichPing-Fing ChiuElia Diego-GarcíaPing-Jyun SungEliana Badiale FurlongPiotr DobrowolskiEliana L. Faquim-MauroPiotr GolińskiElias AlisaacPiotr IwaniukÉlio SucenaPiotr Jan JedziniakElisa CairrãoPiotr JedziniakElisa RussoPiotr MorasiewiczElisa Yoko HirookaPiotr WitkowsiElisabeth VargaPiyush PandeyElisabetta BigagliPo-Cheng HsuElisabetta CapraiPradeep KumarEliza MatuszewskaPrasath ManogaranElizabeth FernandesPrzemysław KosobuckiElsa BonnaféQian YangElsa Leclerc DuarteQianqian ZhaiElvira CrescenziQiao YangElżbieta PatkowskaQiumin LuElżbieta PytlarzQurban AliEmanuela GregoriRachel ClareEmelyn SalazarRadu MineaEmilia BonarRadu RacovitaEmilie LanceRafael CarballeiraEmilio SalgadoRafael J. BorgesEmmanuel LemichezRafael Stuani FlorianoEnrico BraccoRafal OgorekEqram RahmanRaghavendra Rao PasupuletiEric A. AlbrechtRahul GauttamEric JohnsonRahul KumarErica PackRaimundo Jiménez BallestaErnani PintoRaj KumarEsperanza Rivera-De-TorreRajendra AcharyaEstéfani García-RíosRajendran VelmuruganEster Garcia-CelaRajesh P. ShastryEstuardo Lopez-VeraRajiv ReebyeEszter Fliszár-NyúlRalf GerhardEugénia De AndradeRama Rao TataEugénia GallardoRamona D’AmicoEuikyung KimRan ZichelEustachio TarascoRaquel P. F. GuinéEvangelia LivaniouRaquel SoaresEvgenia GlukhovRaquel TorrijosEwa KowalskaRatnakar TripathiEwa LepiarczykRaul Ferrer-GallegoEwa RopelewskaRavinder KumarEwa Żurawska-PłaksejRavindra ThakkarEwelina KowalczykRebecca CreamerEwelina PatyraRebecca NelsonFabiana SupertiRegiane Cristina Oliveira de Freitas BuenoFabien DarfeuilleRegiane Rodrigues Dos SantosFabio Di NardoReinhard ZelenyFabio GosettiRenata PilkaityteFabrizio AnniballiRené KöffelFabrizio Dal PiazReut FalachFabrizio DamianoReuven RasoolyFadia YoussefRhodora V. AzanzaFalk ThielemannRiccardo BientinesiFarman Ali KhanRiccardo TelliniFatema BhinderwalaRichard A. MandervilleFatima El KhalloufiRichard BarbanoFatima RizviRichard HarrisFedor NovikovRichard J. StarkFelipe Penagos-TabaresRichard UwieraFélix A. UrraRimma KalinaFelix Javier Jiménez-JiménezRobert KosickiFengjie SunRobert L. JarretFeng-Yih YuRoberta Di LecceFerenc BagiRoberta FuscoFernanda CosmeRoberto CastiglioneFernanda PortaroRoberto ChiarelliFernando RamalhoRoberto CiarciaFilippo GaviRodolfo IppolitiFilippo RossiRodríguez-Carrasco YelkoFiorella TonelloRoger K. KhouriFlemming AndersenRohitash JamwalFlorian GesslerRomana Roje-BusattoFlorian SchröperRomeo T. CristinaFranca RossiRomeo Theodor CristinaFrancesc Xavier AvilésRonald B. BrownFrancesca DebegnachRonald JennerFrancesco BonoRonel BiréFrancesco CacciolaRosa BellavitaFrancesco EspositoRosa Elena CardozaFrancesco InchingoloRosario NicolettiFrancesco MartelliRossana MorabitoFrancesco TiniRussolina Benedeta ZingaliFrancis VerbekeSabina PodlewskaFrancisc BodaSabine PellettFrancisco CruzSabino PachecoFrancisco E. NicolasSachit AnandFranck MartinSafa OufensouFranco MutinelliSajjan GroverFrederick Matthew KuhlmannSakthivel VaiyapuriFuchang LiSalvatore Giovanni De SimoneFujiang HouSamuel E. AdunyahFulvio CiriacoSamuel EspinoFuminori KatoSamuel K. MutigaFurong TianSamy SelimGabriel AlcobaSandeep KondakalaGabriel HancuSandra HilárioGabriel Navarrete-VazquezSang-Han LeeGabriel PlavanSanja DabelicGabriele DonatiSantiago GutierrezGabrijela Tavcar KalcherSara BonettaGalina NikolovaSara CunhaGanesh BavikatteSara DamianoGang HuaSara De MartinGarbiñe OlabarríaSara MarinelliGarcía-Mendoza ErnestoSara RicciGeoffrey MasuyerSara TombelliGeorge N. HotosSaranya PoapolathepGeorge William KajjumbaSatoshi NagaiGeorgiana DeakSaúl Gómez-ManzoGeorgios A. PappasSchyler A. EllsworthGerald CohenSebastian GnatGeraldo S. MagalhãesSebastian UlrichGerardo CorzoSeiji InoueGerhard AdamSergey KozlovGerhard LitscherSergio NavarroGermain Sotoing TaïweSeth PincusGeromy G. MooreSeungjun LeeGian Luca MarellaShahram MahmoudiGiancarlo PerroneShahzad IqbalGianni GalavernaShahzad Zafar IqbalGilad YahalomShaleen B. KorchGiovanni BocciaSharath Chandra GaddelapatiGiovanni CochettiShauna Farr-JonesGiovanni PalleschiSheng LiGiulia MarroneSheng ZhangGiuseppe BadagliaccaSheng-Nan WuGiuseppe CosentinoShengwen Calvin LiGiuseppe DionisioShigeki KamitaniGiuseppe EspositoShigeru SatoGiuseppe Massimino CocuzzaShirin AhmadiGiuseppe MecaShogo YamakiGiuseppe PascarellaShozo YanoGopal SinghShu WakinoGoran GajskiShunbo LiGoran KišShuowei CaiGoran MitulovicSiddhihalu W. A. HimayaGraeme C. ClarkSiemowit MuszyńskiGreg BeilhartzSijun ZhengGregorio VonoSilverio RotondiGremski Luiza HelenaSilvia CappellozzaGreta GaianiSilvia DossenaGretty VillenaSilvia LaiGriet GlorieuxSilvia LiscianiGrzegorz OnikSilvio TundoGuangbo GeSilvio VeigaGuan-Jhong HuangSimon ClarkeGudula SchmidtSimon G. PatchingGuillermo De La RosaSimone BacchiocchiGuillermo MoreirasSiska CroebelsGuillermo Tellez-IsaiasSkylar Y. CooperGünter BraderSlaven ZjalićGurdal SahinSlavica StankovićGuru Raghavendra ValicherlaSlawomir GonkowskiGustaaf M. HallegraeffSofia AgriopoulouGustavo FernandesSofia Cancela DuarteGuy RostokerSofia MavrikouGwenaelle Lavison-BompardSoledad Pizarro-SánchezH. J. Van Der Fels-KlerxSom DevHa Ryong KimSonia MarinHaiyuan CaiSoobin JangHang-Kin KongSouren PaulHan-Jia LinŠpela ŠalamonHannu J. KorhonenSpiridon MantzoukasHao-Ching WangSrinivasan RamalingamHaq Abdul ShaikStanislas BatailleHarald HefterStanislav TrdanHarley L. WorthyStanislaw OłdziejHaya Lorberboum-GalskiStefan KahlertHayssam M. AliStefanie GierHeba SahyonStefano RavaioliHector M. Mora-MontesStefano RealeHedvig BölcskeiStelian Sergiu MaierHee-Soo ParkStephan PflugmacherHefei ZhaoStephane RetyHei-Sung KimStephen A. KlotzHelena OstolazaStephen MelvilleHelvi Heinonen-TanskiStephen R. JohnsonHema Prasad NarraSteven J. FruchtHernández HerreroStoicho StoevHester-Mari BurgerSuguru YamamotoHideaki TsugeSumeet Kumar SinghHidetoshi InagakiSun ImHiroki ShibataSunita KeshariHiroki ToyodaSurajit BhattacharjyaHiroshi ShimadaSuresh K. VermaHiroyuki TsuchiyaSusana MendesHoang Thi Minh HienSusana Santos BragaHolger BarthSusumu OhyaHongtao LeiSuzana K. StrausHongwei LiuSuzuki ToshiyukiHoon KimSvetlana KamzolovaHorst PosthausSvetlana TomićHossain Mohammad Arif UllahSvetlana V. GuryanovaHossam El-BeltagiSvetoslav TodorovHossein AhangariSwapan ChakrabartyHouda BerradaSwarnalatha MoparthiHrvoje BudinčevićSweta VangavetiHrvoje PavlovićSylvia SoldatouHsiao-Wei LiaoSylwia Śliwińska-WilczewskaHsin-Tang LinSzabo Zoltan-IstvanHsu Yang LinSzemán-Nagy GáborHuahua YuTafuri AlessandroHuan ZhangTakahito ChijiwaHui Wen FanTakashi YadaHui-Hua HsiaoTakehiro SuzukiHung N. MaiTakemi OtsukiHyang Sook ChunTakeshi TsumurayaHyun Jung LeeTakeya KittaHyung-Jin LeeTamara TavoloniHyun-Woo ParkTamas KoszegiIan R. FalconerTaner SarIan ScottTânia VidalIbrahim ElsohabyTanja Žuna PfeifferIbrahim KhalifaTao LanIda Sigueko Sano MartinsTapani MatttilaIghli Di BariTariq MukhtarIkbal Agah InceTatyana PasechnikIlaria D’AcquaricaTeodora TelecanIlias S. TravlosTeodorico De Castro RamalhoIlona SadokTerence L. BradshawImad KansauTeresa JuanIngars ReinholdsTeresa M. VasconcelosIngrid CipakovaTeresa MougaIngunn SamdalTerrence J. RavineIoana GomoiuTeshamae S. MonteithIoana Marina GrinţescuThalita CaladoIoannis A. GiantsisTheo TasoulisIoannis VamvakarisTheodore WeinIon SanduTheodoros EleftheriadisIrena IvshinaTheodoros MavridisIrfan A. RatherTheodoros Varzakasİrfan ErolThérèse MalliavinIrina Gabriela CaraThomas F. MurrayIrina GladkikhThomas ProftIsabel Bermudez-DiazThorsten KucziusI-Shiang TzengTiago NazarethIsidora RadulovTianfang WangIsidro A. PérezTibor FülöpIva SovadinováTihana KurtovićIvan PecorelliTiina RekandIvana GiangriecoTim FosterIvana GobinTimothy SatterleeIvana PolišenskáTimur Yu MagarlamovIvanka TenevaTina NoutsosIvanka TsakovskaTobias BäumerIwona KowalskaToby SananIwona Wojtasik-KalinowskaTom HillIzabela ZakrockaTomas TorrobaJacob GalanTomasz BlicharskiJacqueline Almeida Gonçalves SachettTomasz GóralJadwiga ManiewskaTomasz JędrzejewskiJadwiga WyszkowskaTomasz OszakoJaffer AlsolaissTomasz StompórJaime Andrés PereañezTomasz SzablewskiJaime Marcial-QuinoTomohisa OgawaJames J. ValdésTomoko KhodaJames L. KlotzTomoya NishinoJames La ClairTong ChenJames WainainaToyohiro HamaguchiJanaviciene SigitaTran Dang XuanJang-Seu KiTsz Tin YuJanja BabičTuba EsatbeyogluJan-Peter HildebrandtTünde PusztahelyiJanusz DabrowskiTzi Bun NgJari IntraTzong-Yuan WuJarosław SławekUlrich OrdaJasenka Gajdoš KljusurićUrban FietzekJasna BosnirUrszula WachowskaJason MacranderUte BotzenhartJason W. MarionUwe WalterJaume TorresV. Yeka ZhimoJay W. FoxValentina CecariniJeannette KluessValeria CostantinoJeffrey SilberzweigValeria GagiuJelena KruljValeria TerziJelena MandičValerija DunkićJelena MuncanVan Nguyen TranJelka PleadinVance Girard NielsenJenni StockanVanessa MoreiraJennifer T. GrierVasia S. MavrogianniJen-Tsung ChenVeronica Alexandra AntipovaJer-Ming ChangVerónica Carrasco-SánchezJernej ŠribarVesa Cosmin MihaiJeroen KoolVictor KagotJerome MontnachVictor M. JimenezJesús M. González-JartínVictoria ManeuJesús M. MercadoVidal Haddad JuniorJesus Martín-GilVijaya Anand ArumugamJesus Morón-LópezViktoriya S. SidorenkoJesus Olivero-VerbelVinay ShivannaJian ShiVincenzo MarcotrigianoJianhong XuVioleta G. TruscaJianmei YuViorica Railean-PlugaruJian-Yong WuVitalii TimofeevJiaying ZhuViviana Toro-IbacacheJi-won RyuVladimir DvoretskyJoachim BeigeVladimír FrištákJoachim SurmVladislav FomenkoJoan Manuel Rodríguez-DíazWajid RehmanJoana DamásioWaldemar KazimierczakJoanna GiebultowiczWalden Emil Bjørn-YoshimotoJoanna MatysiakWang-Chou SungJoão Guilherme De Moraes PontesWayne HodgsonJoaquin Gomis-CebollaWayne LitakerJoaquín María CamposWayne Thomas ShierJocelyn SmithWei-Chyou WeiJoel JamesWei-Dong YangJoerg BlessmannWeiming OuyangJohannes WöstemeyerWence WangJohn KapolosWenchao LuJohnna A. BirbeckWen-Chieh SungJoji OtakiWenhui GUJolanta WawrzyniakWen-Sen LeeJolita RadušienėWensen LiuJonathan PuddickWen-Sheng LiuJordi MolgóWenxiao JiangJörg RadermacherWidiastuti SetyaningsihJörg WisselWiesław BielasJorge A. GuimarãesWilliam D. PickingJosé AguileraWilliam MoarJosé Antonio RodríguezWilliam PrimackJosé Ascención Martínez ÁlvarezWilliam S. LawrenceJosé Fernando Huertas-PérezWilliam SchwanJosé Javier Fernández CastroWilliam ZaragozaJosé Javier Zamorano-LeonWillibald WonischJosé LeaoWilman CarrilloJosé Luís Martín-BarrasaWilmer PereraJosé Luis Rodríguez GutiérrezWioletta BielJosé M. FerrerasWissem MnifJosé Martínez-HernándezWojciech KrztońJosé Miguel FerrerasWojciech WakulińskiJosé Rafael De AlmeidaWulf-Dieter MollJosefa TolosaXesús FeásJosep MercaderXiangping ChuJoseph BarbieriXianjiang LiJoseph Calvin KouokamXiaoli ShenJoseph JankovicXiao-Ping LiJosephine WeeXiaozhen MouJosko BozicXie BingJovana KosXingyi JiangJózsef KissXin-Tang LinJuan BlancoXiulian SunJuan BlasiXun WangJuan Carlos Ángeles-HernándezYacine NiaJuan Carlos MoltóYacov ReismanJuan Carlos Moreno-PirajánYajun YangJuan FerréYang BiJuan Luis Jurat-FuentesYang YushunJuan Palacios-OrtegaYanhong DongJuan-García AnaYanwen XiongJudit TelegdiYasuka MatsunagaJulian Balanta-MeloYasushi HiraokaJulian NaglikYasuyuki NagasawaJuliana Pavan ZulianiYazine MahjoubJuliana ScianiYelena Valentinovna LikhoshwayJung-Suk SungYen Chia ChenJunhong WeiYien Che TsaiJunsei MimuraYing JiaJunsei TairaYiqun DengJürgen FritschYongcai LiJürgen PannekYongming BaoJustin SchmidtYoonja KangJustina PradaYoshiko Sugita-KonishiJustyna SzulcYu CaiKai ZhangYuan-Bin ChengKaio Cesar Chaboli AleviYuan-Hong JiangKalaivani ParamasivanYucheng XiaoKalkunte SrivenugopalYu-Hsiang KuanKamalakar ChatlaYuhsuan TsaiKamila Rybczyńska-TkaczykYu-Huei LiuKandasamy SaravanakumarYuichi TsuchiyaKarla De Castro Figueiredo BordonYukari MiseKarolina Anna Wojtunik-KuleszaYuki TakaiKatarzyna Kiliś-PstrusińskaYukihiko MatsuyamaKatarzyna ŚliżewskaYuliia KochiieruKatarzyna Sykłowska-BaranekYuliya KorolkovaKateřina BreiterováYumiko KomoriKatja KolleweYury BelyiKatja SelbyZakaria TazartKatsuhiro KonnoZakhar O. ShenkarevKatsuya GomiZala KolencKavita SharmaZapryana R. DenkovaKay PerryZdeněk KejíkKazuhiko MaedaZdeněk TůmaKeerthi S. GurugeZdeňka SvobodováKeisuke EkinoŽeljko JakopovićKen TeterZhaojiang GuoKenneth I. OnyedibeZhaowei ZhangKent M. ReedZhen WangKeshav KumarZheng WangKevin BohannonZhen-Ying WangKe-Vin ChangZhihao SunKhalil Saad-AllahZhiyong GongKhan Muhammad ImranZhi-Yong GongKianoosh Khosravi-DaraniZhiyong ZhaoKihwan ChoiZhonghua LiuKim StanfordZiarno MałgorzataKirill AntonetsZoltán BratekKo-Hua TsoZoran MarinovicKonrad HusZuzana AndrejčákováKou KitabayashiZuzana Hruska


